# The Book World of Medicine and Science

**Published:** 1898-05-28

**Authors:** 


### THE BOOK WORLD OF MEDICINE AND SCIENCE.

THE REPORT OF THE ROYAL COMMISSION ON VACCINATION. A REVIEW OF THE DISSENTIENT STATEMENT. By JOHN McVAIL, M.D. (London: P. S. King and Co., 1898. Pp. 128, 8vo.)

This trenchant criticism of the minority report, signed by Dr. Collins and Mr. Picton, was read before the Epidemiological Society of London a year ago. The society has wisely decided to issue it in a form generally accessible. Dr. McVail has attacked Dr. Collins and Mr. Picton on their own ground, and routed them all along the line. After proving, from their own mouths, that they admit vaccination as a mode of stamping out small-pox and hold the difference between themselves and the majority of the Commission to be one of degree only, Dr. McVail very neatly convicts them of various gross inconsistencies. For instance, we find in the dissentient report a statement that at the beginning of this century 80 per cent. of the population was "protected" by previous small-pox. Yet in the *Contemporary Review* Mr. Picton has declared that only a minority were so protected. The attempt of Dr. Collins to prove Woodville's lymph to have been variolous completely breaks down under criticism. And here again the dissentients are placed between the horns of a dilemma. They claim that the success of much early "vaccination" was due to the fact that the process was really "variolation." Yet in another paragraph they declare that the diminution of small-pox at the time referred to was due to diminished inoculation; in other words, they state (1) small-pox diminished because of free variolation, (2) small-pox diminished because variolation was abandoned. The obvious fallacy involved in calculating attack-rates amongst vaccinated and unvaccinated persons, from figures drawn from the records of the London Small-pox Hospital, is thoroughly exposed. This hospital admits cases which are nearly all drawn, as Dr. Collins should have known, from the adult and vaccinated classes. Dr. McVail indulges incidentally in some severe and instructive remarks on Professor Crookshank's methods of evidence, and makes an excellent point out of the diametric opposition of Dr. Creighton's views on vaccinal syphilis to those of his colleagues on the Anti-Vaccination League. The statistical portion of Dr. McVail's essay, and his method of dealing with the question of isolation, are convincing and powerful. Dr. McVail does not, in this essay, set out to prove the case for vaccination. But he carries the war into the enemies' camp, and by exposing the utter worthlessness of all the arguments that the most ingenious and able anti-vaccinists have brought forward, furnishes his own party with a perfect armoury of controversial weapons of precision.

A SYNOPSIS OF THE BRITISH PHARMACOPŒIA, 1898. Comprising Concise Notes on the Changes, Particulars of New Preparations, Tables of Doses, &c. Compiled by H. WIPPELL GADD. (London: Baillière, Tindall, and Cox.)

We have just received this very handy little book, which contains in the form of alphabetically arranged tables what the preface calls "a complete synopsis" of the new pharmacopœia. There is no doubt about it being "complete," for although it is but a tiny book it apparently goes straight through the pharmacopœia, and gives under each substance the name, the character, the dose (both according to imperial and metric measure), and remarks, among the latter being comments as to strength, as to composition, and as to preparations derived from it—all in fact that most people want to know. How the Treasury will like the bread to be taken out of their mouths by the publication of handy little volumes of this sort we cannot say, but certainly the book before us seems to contain all that the ordinary practitioner will care for. No doubt he could not make up the preparations, but who wants to make up the preparations? That is a matter for the chemist, who must, of course, buy the official book, but the prescriber will, we fancy, be content with the synopsis.

RESPIRATORY EXERCISES IN THE TREATMENT OF DISEASE. By HARRY CAMPBELL, M.D.Lond. (London. 1898. Baillière, Tindall, and Cox. Pp. viii. 200. Demy 8vo.) Price 7s. 6d.

Dr. Campbell's latest work has perhaps a wider scope than the title indicates. It deals at some length with various physiological aspects—notably the muscular—of respiration; with methods of respiration practised by vocalists, and other topics. The latter portion of the work it is which is devoted to the discussion of the effects of respiratory exercises in health and disease. Dr. Campbell's views, if not always very novel, are eminently rational and well considered. The amount of actual "exercise" involved by much speech is effectively pointed out; and the physiological justification of "a good cry," of sighing, and of yawning is insisted on. Respiratory exercises are, perhaps, of most therapeutic value in heart disease; but Dr. Campbell employs them with success in phthisis, dyspepsia, and neurasthenia. It is difficult, however, to see what advantage, in the latter cases, respiratory exercises have over massage and other "exercises." In heart disease, on the other hand, the effects of respiratory exercise, as in Oertel's treatment, are specific. A very excellent chapter is that in which Dr. Campbell deals with some of the still obscure and unsolved problems of emphysema. Dr. Campbell's book will well repay perusal; it is carefully and accurately written, and supplies considerable matter for profitable reflection.

LEXIQUE-FORMULAIRE DES NOUVEAUTÉS MÉDICALES. By Professor PAUL LEFERT. (Paris: J.-B. Baillière et Fils. 1898. Pp. 336.) Price 3 frs.

A most useful little book, the exact likeness of which we do not seem to possess in English. By aid of clear type and thin paper it has been found possible to compress an immensity of information into a very small compass. Its title is its description, for it is a dictionary of disease and treatment as well as a formulary of medicines. The object has been to arrange alphabetically for easy reference those recent additions to our knowledge which have not yet got into the text books but remain for the most part buried in various journals and periodicals which are not always easy to lay hands upon. We do not say that whatever is new has been welcomed, for the book shows signs on every page of careful selection and arrangement; but most things that are new are mentioned, all sorts of subjects following one another with the extremest impartiality. New diseases, new groups of symptoms—syndromes to which new meanings are attached—new remedies, and especially new operative proceedings, are all described, and wherever drugs are dealt with formulæ for their administration are appended. We are much pleased with this book, which has been most carefully done.

WHO'S WHO 1898. Edited by DOUGLAS SLADEN. (London: Adam and Charles Black. Pp. 823.) Price 3s. 6d. net.

It is late in the year to notice an annual publication like "Who's Who." Nevertheless the very fact that we did not deal with it earlier enables us to speak to its merits with all the confidence which is born of daily familiarity with them. As a means of saving the worry of uncertainty in regard to all sorts of little things that everybody is supposed to know and that everybody forgets, "Who's Who" is an admirable companion on the writing table.

